# Hyperprogressive Disease in Anorectal Melanoma Treated by PD-1 Inhibitors

**DOI:** 10.3389/fimmu.2018.00797

**Published:** 2018-04-19

**Authors:** Marjorie Faure, Philippe Rochigneux, Daniel Olive, Sébastien Taix, Isabelle Brenot-Rossi, Marine Gilabert

**Affiliations:** ^1^Medical Oncology Department, Paoli-Calmettes Institute, Aix-Marseille University, Marseille, France; ^2^Team Immunity and Cancer, Centre de Recherche en Cancérologie de Marseille (CRCM), INSERM, U1068, CNRS, UMR7258, Paoli-Calmettes Institute, Aix-Marseille University, Marseille, France; ^3^David Geffen School of Medicine at University of California Los Angeles, Los Angles, CA, United States; ^4^Immunomonitoring Plateform, Paoli-Calmettes Institute, Aix-Marseille University, Marseille, France; ^5^Pathology Department, Paoli-Calmettes Institute, Aix-Marseille University, Marseille, France; ^6^Nuclear Medicine Department, Paoli-Calmettes Institute, Aix-Marseille University, Marseille, France; ^7^Centre de Recherche en Cancérologie de Marseille (CRCM), INSERM U1068, CNRS UMR 7258, Parc Scientifique et Technologique de Luminy, Paoli-Calmettes Institute, Aix-Marseille University, Marseille, France

**Keywords:** hyperprogression, melanoma, immune checkpoint inhibitors, immunomonitoring, anti-PD1

## Abstract

The 5-year survival rate of primary anorectal malignant melanoma is less than 20%. Optimal treatment of this condition remains controversial regarding locally disease, and whether any preferential survival benefit arises from either abdominoperineal resection or wide local excision remains unknown. The majority of patients progress to metastatic disease, and for decades, the use of chemotherapies, such as platines or dacarbazine, has been advocated to improve overall survival. The therapeutic use of new checkpoint inhibitors in a variety of trials has provided evidence for an antitumoral effect of PD-1 and/or CTL4 inhibitors in mucosal melanomas, but these treatments must still be further evaluated. Some anecdotal occurrences of rapid progression [i.e., hyperprogressive disease (HPD)] while using these immune agents have been described, suggesting potentially deleterious effects of these drugs for some patients. We report a 77-year-old male metastatic anorectal melanoma patient presenting with HPD over 2 months of a PD1 inhibitor treatment course and document this HPD blood phenotype.

## Introduction

Anorectal malignant melanomas (ARMM) comprise approximately 1% of all melanomas and approximately 0.5–2% of all anorectal malignancies (1). It affects the anal canal, rectum, and intermediate sites in equal proportions (2). The median overall survival after diagnosis is between 8 and 19 months (3), and the 5-year survival is 20 and 0% in cases of locoregional resectable disease and advanced disease, respectively (4). A poor overall survival in ARMM is associated with male gender, perineural invasion, infiltration depth of the rectal wall, lymph node metastasis and distant metastasis. Conventionally, to control the local disease, therapy consists of a complete surgical resection of the tumor. This can be done by means of sphincter-sparing wide local excision or abdominoperineal resection (APR) (5). Radiotherapy may be used to enhance regional control but has no impact on overall survival (6). Because it is an aggressive malignancy, most patients become metastatic after a few months, and for decades, advanced ARMMs were commonly treated by systemic chemotherapies (7), such as cisplatine, vinblastine, and dacarbazine, or by immune agents such as interferon alpha-2b (IFN) and interleukin 2; however, these treatments have low effectiveness on response and survival.

Although conventional chemotherapies have shown only a minor benefit to patients with advanced melanoma (8), therapeutic immune antibodies against programmed cell death receptor 1 (PD-1) and programmed cell death receptor ligand 1 (PD-L1) have demonstrated a significant and durable response, either in front-line therapy or in subsequent therapies (9). However, the efficacy of PD-1 blockade in patients with biologically distinct melanomas arising from mucosal surfaces has not been well described, and data are still lacking (10).

Notably, anecdotal evidence of rapid disease progression in patients treated with anti-PD-1/anti-PD-L1 monoclonal antibodies (mAbs) has been reported (11). This was shown by a French review of the tumor growth rates in 131 patients upon treatment with anti-PD-1 therapies, which revealed that 9% of patients developed hyperprogressive disease (HPD), characterized by accelerated tumor growth (12).

Here, we report a case of ARMM treated by a new immunologic therapy PD-1 inhibitor, experiencing HPD over 2 months of treatment, leading to rapid death. We then present the HPD blood phenotype analysis and discussion.

## Case Report

**Figure 1:**
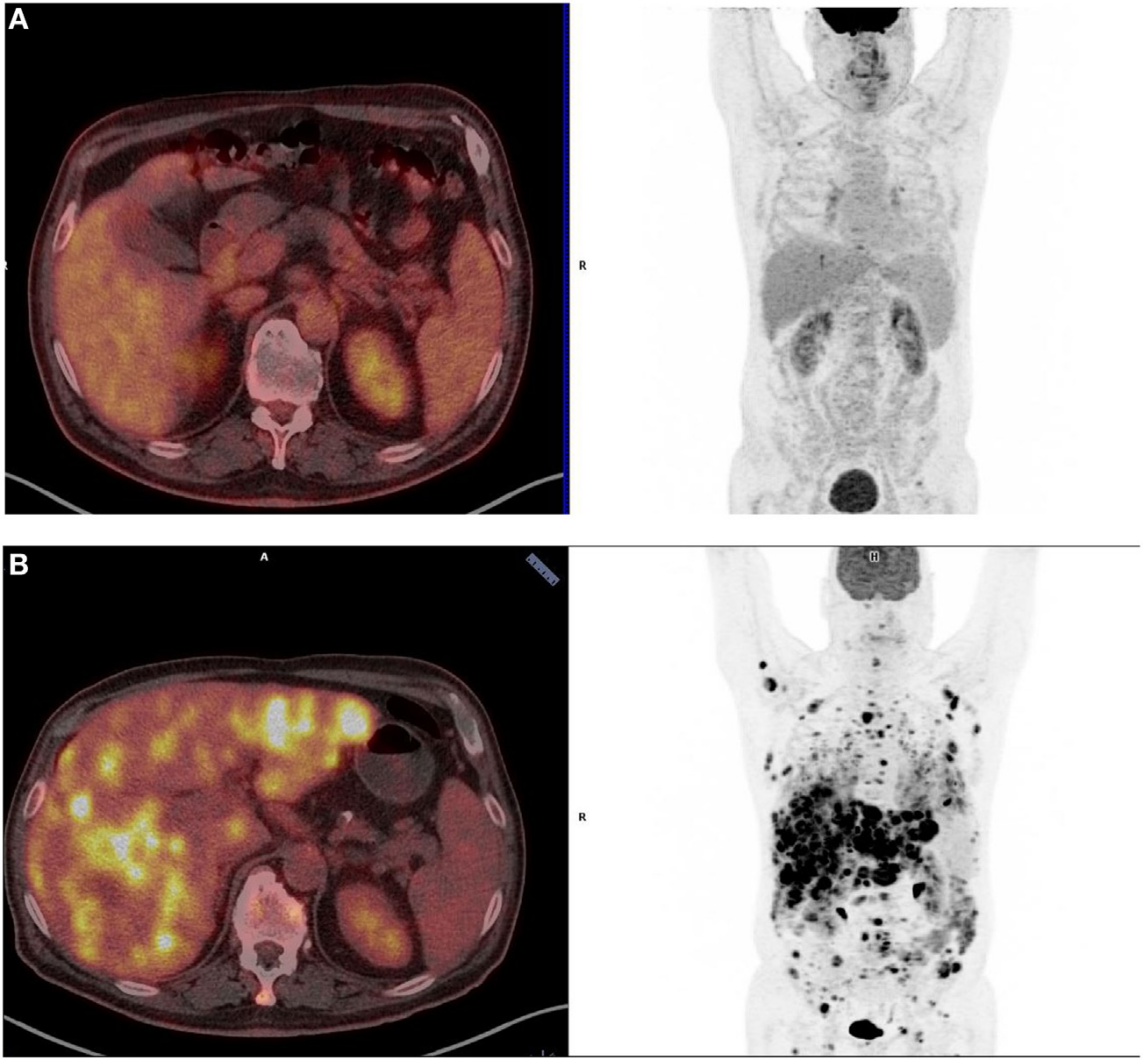
Positron-emission tomography (PET)-scanner imaging at baseline **(A)** and after three cycles of pembrolizumab **(B)**. **(A)** Patient’s baseline PET scan (December 2016): only four infracentimetric hypermetabolic lung nodules are visible [standardized uptake values (SUV) from 2.2 to 5.5]. No evidence of distant metastases elsewhere. **(B)** Patient’s PET scan after three cycles of pembrolizumab (May 2017): diffuse and multiple hypermetabolic lung, liver, subcutaneous tissue, and peritoneal lesions (SUV from 3.5 to 17).

A 77-year-old white male presented sporadic rectal bleeding in April 2016. Rectal examination revealed a mass in the lower rectum. A broad-based rectal polyp measuring 10 mm and located approximately 6 cm from the anal verge was seen on colonoscopy, and a transanal polypectomy was performed. Histopathology diagnosed a 3.3-mm anorectal melanoma with positive stains for Melan A on immunohistochemistry, ulceration, high mitotic index, and inadequate positive resection margins (pT3bR1). Other malignant primary sites and distant metastases were ruled out. The patient underwent revised surgery that consisted of APR with bilateral inguinal lymphadenectomy, following the consensus of the interdisciplinary tumor board. Surgery and recovery were uneventful. The final tumor stage was pT3bN0 (0/15) M0R0, stage IIb (UICC). No perineural invasion was revealed. Neither BRAF nor KIT mutations were identified on genetic analysis. No adjuvant treatment was given according to international guidelines. The follow-up consisted of alternately brain + thoraco–abdo–pelvic Computed Tomography (CT) and positron-emission tomography (PET) scan, every 3 months. Six months later, in December 2016, the thoracic CT scan revealed four new infracentimetric lung nodules that were confirmed to be hypermetabolic mild positive on PET scan (standardized uptake values from 2.2 to 5.5). There was no evidence of other extrapulmonary metastatic spreads (Figure 1A).

The patient was healthy with Eastern Cooperative Oncology Group 0 and no weight loss. As conventional chemotherapies are mostly inefficient, and based on encouraging results regarding patients with mucosal melanoma enrolled in first line therapy PD-1 inhibitor trials, a tumor board council validated a treatment course of pembrolizumab. The patient was given pembrolizumab 200 mg IV, over 30 min, every 3 weeks (D1 = D21) from February to April 2017 (3 injections), with no specific side effects except for fatigue grade 1. However, in May 2017, the patient newly presented a right chest pain and a rapid deterioration of the general status. A PET scan revealed the emergence of multiple new centimetric hypermetabolic lung spots along with increased size and glycolic activity in the previously identified nodules that were associated with multiple new hypermetabolic liver, subcutaneous tissues, and peritoneal lesions (Figure 1B), and showed a rapidly progressive disease over 2 months.

We performed blood tests to biologically document this hyperprogressive (HP) patient, but he refused new tumor biopsy. Immunophenotyping analyses by flow cytometry (BD Fortessa^®^/FlowJo software^®^) were performed from peripheral blood mononuclear cells (PBMC) based on a Ficoll density gradient after the third injection of pembrolizumab. As no other patient samples were available, we compared the sample to healthy donors (HD).

**Figure 2:**
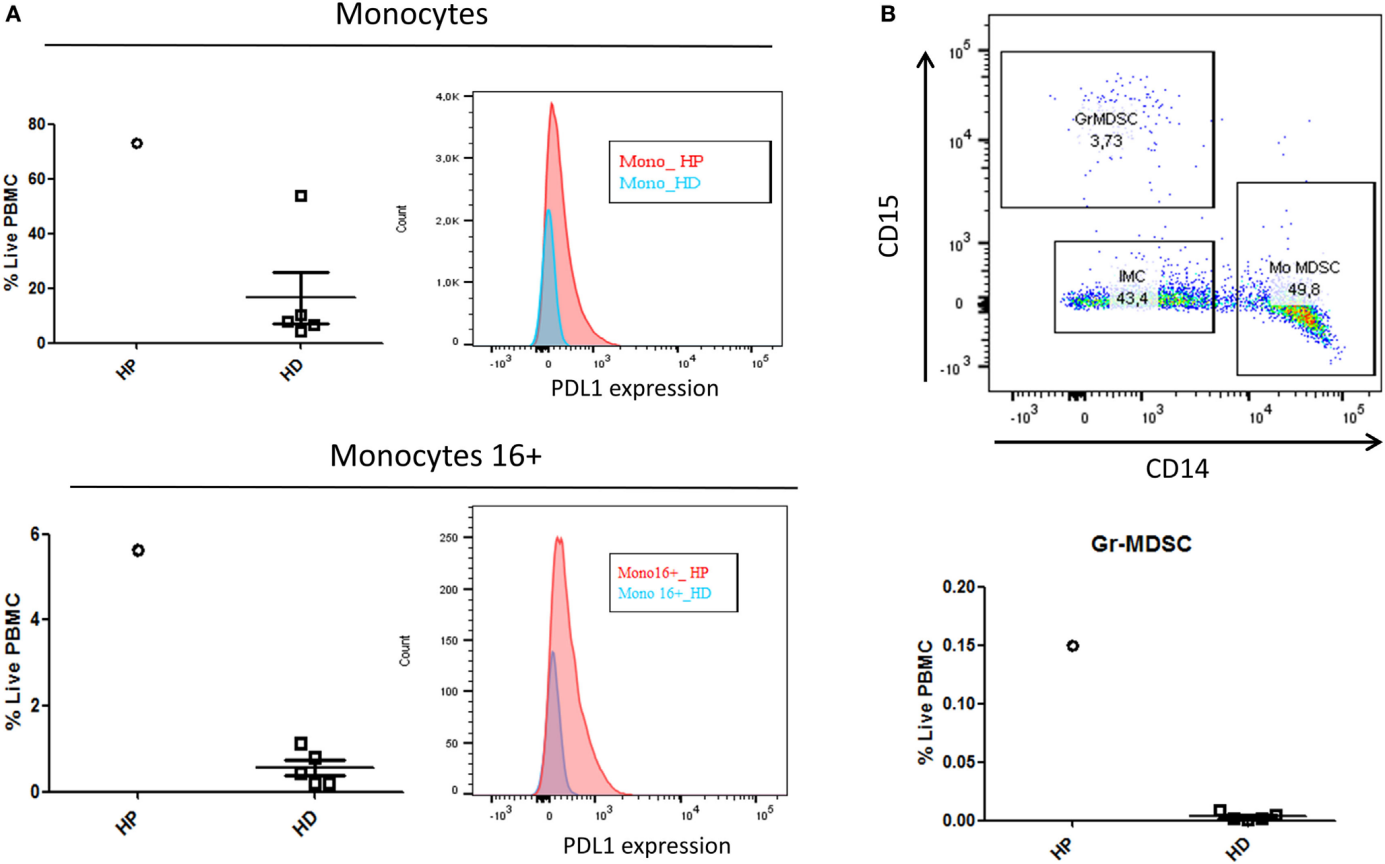
Flow cytometry analysis of myeloid cells comparing the HP with five HD. **(A)** Higher proportion of monocytes (HLADR^+^ CD33^+^, CD14^+^) and CD16^+^ monocytes (HLADR^+^ CD33^+^, CD14^+^ CD16^+^) in HP compared to the HD (pro-inflammatory cells). **(B)** Increased median fluorescence intensity of PDL1 in monocytes/CD16^+^ monocytes, and the more important percentage of the immunosuppressive population of Gr-MDSC in the HP patient compared to HD. Abbreviations: HP, hyperprogressive patient; HD, healthy donors; Mono, monocytes; Mono16+, monocytes 16+; GrMDSC, granulocytic myeloid-derived suppressor cells; Mo-MDSC, monocyte-derived suppressor cells; IMC, immature myeloid cells; PBMC, peripheral blood mononuclear cells.

In the myeloid cells analysis presented in Figure 2, the HP patient had a higher proportion of monocytes (HLADR^+^ CD33^+^, CD14^+^) and CD16^+^ monocytes (HLADR^+^ CD33^+^, CD14^+^ CD16^+^) compared to the HD (Figure 2A). Furthermore, the patient’s monocytes and CD16^+^ monocytes had an increased median fluorescence intensity of PDL1 compared to HD. Additionally, between the live PBMC samples, the percentage of the immunosuppressive population of granulocytic myeloid-derived suppressor cells (Gr-MDSC), defined as CD33^+^/HLADR^−^/CD15^+^/CD14^−^, was increased in the HP patient (Figure 2B).

**Figure 3:**
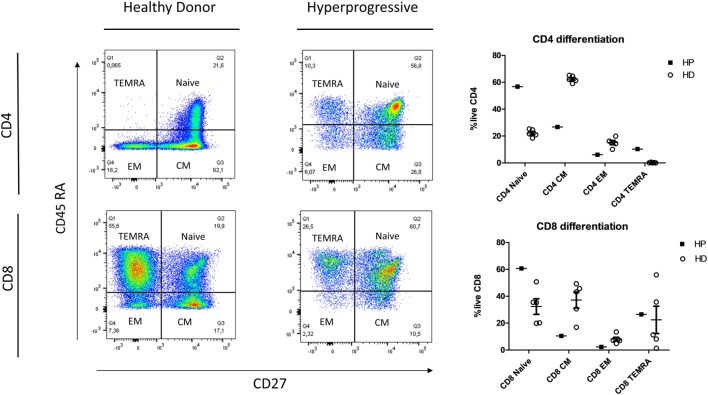
Flow cytometry analysis of lymphoid T cells comparing the HP with five HD. HP showed an increased proportion of naïve CD4/CD8 and CD4 terminally differentiated cells (TEMRA: effector memory CD45RA^+^) and a decrease in memory cells (CM/EM). Abbreviations: HP, hyperprogressive patient; HD, healthy donors; CM, central memory; EM, Effector Memory; TEMRA, Terminally differentiated Effector Memory RA+.

**Figure 4:**
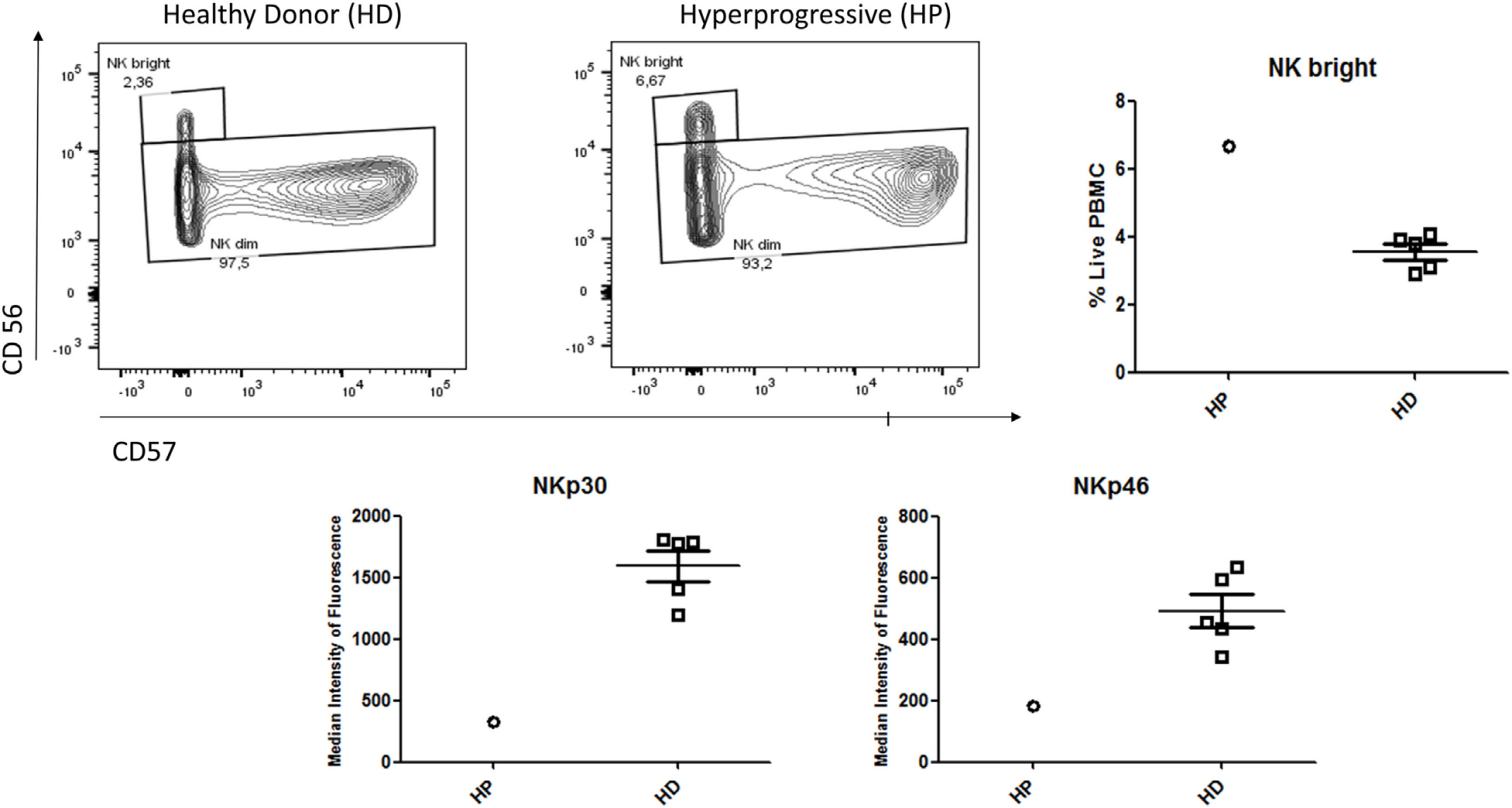
Flow cytometry analysis of NK cells comparing the HP with five HD. Increased proportion of NK bright (CD56^+^ high) cells in HP compared to HD. Conversely, decreased expression of the receptors NKp30 and NKp46 in HP compared to HD. Abbreviations: HP, hyperprogressive patient; HD, healthy donors; NKp30/NKp46, natural cytotoxicity receptors.

In the lymphoid cell analysis presented in Figure 3, the HP patient presented a shift in CD4 and CD8 differentiation, with an increased proportion of CD4/CD8 naïve and CD4 terminally differentiated cells (TEMRA: Effector Memory CD45RA^+^), and a decrease in memory cells (central memory/effector memory). Among natural killer cells (NK cells), the HP patient had an over-expression of NK bright (CD56^+^ high) cells and an under-expression of the receptors NKp30 and NKp46 (Figure 4).

After three cycles of pembrolizumab, one cycle of salvage dacarbazine was given, but it did not bring any clinical improvement. The patient was then treated with best supportive care on a palliative basis. He unfortunately passed away in July 2017.

## Discussion

In recent years, immunotherapy has revolutionized the standard of care of many solid tumors, especially melanomas, with a significant improvement in overall survival for some patients. Despite this progress, recent evidence suggests that treatment with PD-1 pathway blockade therapy may backfire in a subset of patients, leading to rapid tumor development, a response opposite to that expected (13). HPD is related to a marked increase in the tumor growth kinetics (TGK) and defined by a ≥2-fold increase in the TGK ratio (TGK post treatment/TGK pretreatment), according to the Response Evaluation Criteria in Solid Tumors (RECIST 1.1) (12).

Even though the precise frequency of hyperprogression is yet to be established, this topic is far from anecdotal; it concerns 9% (*n* = 12/132) of the multi-tumoral cohort of the Gustave Roussy Institute and 29% (*n* = 10/34) of a multicentric French cohort of head and neck carcinomas under anti PD-L1 therapy (11).

In this case study, initiation of immunotherapy in an elderly patient who had a slow-growing initial tumor led to HPD. These findings are consistent with emerging data from the literature where patients with HPD tend to be male, elderly, with low tumor volume and slow-growing initial disease at baseline (14).

The histological subtype does not seem to predict HPD since there is no difference in the rate of HPD across the different cancers pathologies including melanoma, urothelial, colorectal, ovarian, biliary tract carcinomas, and lymphoma (12). The number and type of previous lines of treatment, as well as PD1/PDL1 expression, are not predictive factors for HPD (12).

Currently, the immunological basis of HPD is unknown. As this concept has been identified only recently, we are the first to describe the immunophenotyping of a patient with HPD. On the one hand, we have described in this patient an increase in pro-inflammatory myeloid cells (monocytes and 16+ monocytes) and a hyperexpression of PDL1 that is coherent with an activated phenotype. Moreover, the presence in our patient of higher MDSC, known to be immunosuppressive due to its inhibitory effect on T cell growth and functioning, could also partially explain the immunoevasion (15). On the other hand, the shift in the differentiation of T cells in the blood (favoring naïve and TEMRA instead of memory cells) may be related to the tumoral trapping of CD4/CD8 memory cells or death due to hyper-stimulation. Altogether, these elements suggest that the patient is facing an inefficient inflammatory response rather than a tumoral-targeted T cell response. As described previously in the literature, this patient may have had an imbalance between T cell reinvigoration and tumor burden (16).

In this study, some limitations should be considered. First, the sample size was obviously small. Second, the dynamic changes in the levels of circulating cells before and after treatment is lacking, and basal samples should be collected in future studies, rather than using HD as a control. Third, as the patient refused a re-biopsy, we were not able to document the molecular or immunological changes of the tumor after HPD.

Finally, in the field of biomarkers, the clear challenges are to identify biomarkers that will be predictive of a positive clinical response to immunotherapy and target patients at risk of an HPD response, in addition to an age-dependent decrease in immunological competence.

For example, a recent study of 155 patients treated with anti-PD-1/anti-PD-L1 therapy reported that all six individuals with MDM2/MDM4 amplification experienced time-to-treatment failure (TTF) of less than 2 months, and four of the six patients exhibited an HPD response (17). This study further showed that eight of 10 patients with EGFR mutations experienced TTF of less than 2 months, and 2 of 10 demonstrated HPD.

Interestingly, a recent research about anti-PD1 biomarkers using mass cytometry identified the frequency of CD14^+^ CD16^−^ HLA-DR^hi^ monocytes in PBMC before therapy as the strongest predictor of response (18). Our findings, combined to this study, support the idea that monocytes are a key player in anti-PD1 response, notably because of their antigen-presenting functions on CD4/CD8, and their possibility to differentiate into many different cells types (19). However, it is not clear if the dynamics of monocytes during anti-PD1 can predict outcome, and if PDL1 expression in monocytes leads to a functional impairment of CD8^+^ T cells (20).

Further investigations of the HPD response are still needed for the development of decision trees and to guide physicians’ choices to treat a patient with immunotherapies.

## Ethics Statement

Informed consent statement: the patient provided informed written consent prior to the study. The study was reviewed and approved by the Paoli-Calmettes Institutional Review Board.

## Author Contributions

MF and MG designed the research. MF, PR, and MG wrote the paper. PR performed the immunological analysis. MF, PR, DO, ST, IB-R, and MG collaborated on the paper’s conception, reviewed the paper, and approved the final version of the article to be published.

## Conflict of Interest Statement

The authors declare that the research was conducted in the absence of any commercial or financial relationships that could be construed as a potential conflict of interest.

## Abbreviations

ARMM, anorectal malignant melanomas; CM, central memory; ECOG, Eastern Cooperative Oncology Group; EM, effector memory; HPD, hyperprogressive disease; HD, healthy donor; HP patient, hyperprogressive patient; IMC, immature myeloid cells; Mono, monocytes; MDSC, myeloid-derived suppressor cells; PBMC, peripheral blood mononuclear cells; TEMRA, terminally differentiated effector memory RA+.
